# Modified Fine Recycled Concrete Aggregates with a Crystallizing Agent as Standard Sand Replacement in Mortar

**DOI:** 10.3390/ma18174208

**Published:** 2025-09-08

**Authors:** Daniel Suarez-Riera, Luca Lavagna, Devid Falliano, Giuseppe Andrea Ferro, Matteo Pavese, Jean-Marc Tulliani, Luciana Restuccia

**Affiliations:** 1 Department of Structural, Building and Geotechnical Engineering, Politecnico di Torino, C.so Duca degli Abruzzi 24, 10129 Torino, Italy; daniel.suarez@polito.it (D.S.-R.); devid.falliano@polito.it (D.F.); giuseppe.ferro@polito.it (G.A.F.); 2 Department of Applied Science and Technology, Politecnico di Torino, C.so Duca degli Abruzzi 24, 10129 Torino, Italy; luca.lavagna@polito.it (L.L.); matteo.pavese@polito.it (M.P.)

**Keywords:** recycled concrete aggregate, waste recycling, mortar, crystallizing agents, sustainable construction material, circular economy

## Abstract

This study aimed to evaluate mortar performance by substituting part of standard sand with recycled fine aggregates sourced from concrete waste, aiming to assess mechanical properties and durability. Moreover, this study examined the use of crystallizing agents to understand their impact on mortar properties. Four mortar series were prepared with sand substitution percentages ranging from 25% to 100% while adhering to the diverse fraction proportions within the standardized sand particle size distribution. Mechanical results indicate that incorporating recycled concrete sand significantly enhances mechanical properties with respect to standard sand. The study showed the technical feasibility of producing mortars with up to 100% recycled fine concrete aggregate with enhanced compressive strength, albeit requiring higher superplasticizer dosages. The addition of crystallizing agents provided an increase in flexural strength in specific conditions, while they did not provide a significant improvement to compressive strength.

## 1. Introduction

The global economy is facing a significant challenge with its current linear model of production and consumption. A linear economy (LE), also known as the “take-make-waste” model, is a traditional economic model where resources are extracted, processed into goods and products, and eventually discarded as waste after being used [1]. Consequently, LE generates a large amount of waste, which is simply viewed as a by-product of economic growth, including non-biodegradable materials, and consumes finite resources that are being depleted at an unsustainable rate [2,3]. Within this context, the construction sector is associated with the depletion of natural resources to manufacture building materials, consuming up to 40% of global raw materials extracted every year, and generating enormous amounts of construction and demolition waste (CDW) [4,5,6]. Subsequently, there is a growing interest in the circular economy approach to achieve sustainable development through the 3R principle (reduce, reuse, and recycle), creating a closed-loop system where resources are kept in use for as long as possible [7,8].

Various measures are being taken to increase sustainability and to reduce the adverse environmental impact of building materials. Within the cement-based materials, this impact can be mitigated using alternative materials. For example, using recycled aggregates (RAs) from construction and demolition practices in mortar and concrete production can decrease the amount of landfilled waste and reduce the consumption of virgin materials since the increasing amount of produced waste and its disposal step negatively impacts the environment and society. This category of waste represents around 40% of the total solid waste, with a global recovery rate ranging from 20% to 30% [9,10,11]. In the European Union, for example, approximately 3 billion tons of waste are produced each year, with one-third of this amount due to construction and demolition activities [12,13], with an average recovery rate of almost 50%. However, the average recovery rate falls short of the 70% target of the Waste Directive 2008/98/EC set for 2020 [14].

Therefore, in recent years, researchers put a lot of effort into studying the properties of RAs obtained from CDW and its potential application in mortar production. Many papers have shown that RAs can be used effectively in place of virgin aggregates in cement-based materials despite some technical problems, mainly in mechanical strength and workability areas [15,16]. Materials finer than 0.08 mm in the dry mix can be used to assess the workability of mortars, as they alter the water requirement and potential shrinkage of mortars with natural sand, recycled sand, or a mix of them [17,18].

In this context, Stefanidou et al. [19] investigated the use of three different mortars (with hydrated lime, a mix of lime and natural pozzolan, or a mixture of lime, natural pozzolan, and cement) with standard sand (SS), natural sand, or recycled sand (RS) for repair works. They found that adding RS to lime-based mortars, with 1 wt% of superplasticizer with respect to cement, can enhance compressive strength, especially at early ages. This improvement may be due to pozzolanic reactions between lime and the silica constituents of the raw materials in the sand. Braga et al. [20] demonstrated the feasibility of using up to 15% of fine concrete recycled aggregates in mortar production, resulting in an improvement in most of the properties of the reference mortar. Additionally, Neno et al. [21] produced mortars with partial (20% and 50%) and total substitution (100%) of natural sand by recycled concrete aggregates and found compressive and flexural strength improvements at 28 days in all the cases with respect to the standard mortar. Notwithstanding, Ledesma et al. [22] studied the incorporation of RAs obtained from ceramic masonry waste in eco-mortars at 0%, 25%, 50%, 75%, and 100% replacement rates. They found that adding RAs decreased the compressive strength of the eco-mortars by almost 12%. However, this loss in compressive strength was acceptable for non-structural applications. They also noted that using RAs increased the mortars water demand and air content. However, the addition of a superplasticizer effectively increased the workability and strength of the mortars while reducing the water demand. Additionally, some of the authors of this paper studied the influence of washed recycled sand (to reduce the excess fine fraction) as a partial replacement in mortars [23], evidencing that while washing the recycled aggregates does lead to improved results compared to unwashed sand, the overall mechanical performance still does not surpass that of mortars made with standard sand. Furthermore, the washing process does not contribute to the overall sustainability of the system.

Thus, it is well-established that, generally, mortars incorporating RAs tend to exhibit lower mechanical performance compared to those made with conventional aggregates, primarily due to several inherent limitations of RAs, such as increased porosity, higher water absorption, elevated crushing index, the presence of microcracks in the interfacial transition zones (ITZ), contamination, and inconsistent quality [21,24,25]. Notably, the microcracks in the ITZ significantly weaken the recycled aggregates, facilitating the ingress of harmful substances, which react with cement hydration products. This reaction produces expansive compounds, such as gypsum and ettringite, which increase internal stresses and further compromise the structural integrity of the recycled concrete aggregates [26,27]. Consequently, fully saturating recycled aggregates before incorporating them into new concrete is fundamental to guarantee the workability and consistency of the mix. Thus, improving RA microstructural and mechanical properties is of paramount importance to enhance its applicability and usefulness in producing recycled concrete [28,29,30]. The current literature indicates that there are six significant methods to improve the properties of recycled aggregates. These methods can be classified into two groups. Firstly, the “improve by removing” classification encompasses techniques focused on eliminating residual mortar from recycled aggregate, incorporating chemical and thermal processes. Conversely, the “improve by adding” category considers approaches centered on supplementing mineral admixtures, facilitating self-healing mechanisms, promoting carbonation reactions, implementing sequential mixing protocols, and reinforcing through coating and permeation methodologies [31,32,33,34]. In recent years, several recent reviews of the techniques for modifying RAs and selecting the optimal method in function of the different types of concrete are available in [35,36,37,38,39,40,41,42].

From the coating and permeation point of view, few research studies focus on identifying techniques to enhance the microstructural properties of recycled aggregates. However, research in this area is incomplete, leaving an extensive knowledge gap that needs further investigation and filling [42]. In this respect, a way to enhance the properties of RAs through the integration of crystallizing agents’ technology has been identified. This method, denoted as crystalline waterproofing, has garnered widespread adoption within concrete applications. It involves using active substances that react with hydration products or unreacted cement particles in the concrete matrix. These reactions yield supplementary reactants in crystalline form, thereby augmenting the overall performance and durability of the material [43,44,45,46]. These crystallizing agents moreover effectively block off the pores in concrete, decreasing their overall permeability [47,48].

This innovative approach was applied in a limited number of papers to recycled coarse aggregates to fill in pores and cracks [49,50]. Thus, in this work, recycled fine aggregates (RFAs) were soaked in an aqueous solution of a crystallizing agent called Admixplus (AD), a commercial compound from Supershield Italia S.r.l., for the first time, to the best of our knowledge. The porous and cracked nature of the RA powders allows for easy penetration of the AD solution, generating an insoluble crystalline structure inside capillary pores. In hardened concrete, this structure serves as a waterproof barrier against water and chemical agents penetration inside the RA grains. The formation of crystals is also stimulated at a later stage in case water or moisture seeps into the material. It is essential to highlight that the producer keeps the chemical composition of the crystallizing agents strictly confidential.

Therefore, the present work proposes a novel approach for enhancing the environmental and mechanical performances of mortars containing recycled fine aggregates previously consolidated by means of crystallizing agents. This study was conducted by replacing standard sand with treated recycled concrete aggregates at varying rates: 25%, 50%, 75%, and 100%. The focus on mortars is the first step of a broader study which aims at producing concrete with either coarse and fine RAs modified with crystallizing agents.

## 2. Materials and Methods

### 2.1. Materials

The materials used to prepare the mortar specimens were Portland Cement Type I, CEM I 52.5 (Buzzi Unicem, Casale Monferrato, Italy), tap water (W) for mixing, casting and curing, a commercial superplasticizer—MasterEase 7000 (Master Builders Solutions Italia Spa, Treviso, Italy)—(SP), and a commercial crystallizing agent AD produced by Supershield Italia S.r.l. (Torino, Italy) for concrete waterproofing, protection, and enhancing its durability. According to the producer, AD reacts with moisture in concrete and forms an insoluble crystalline structure within the capillary pores, serving as a waterproof barrier against water and chemicals. CEN standard sand, with the specific particle size distribution shown in Table 1 was also used. Finally, RAs were provided by F.G. S.r.l., a construction company based in Turin, Italy, which specializes in environmental sustainability, prominently featuring the extraction and processing of both natural and recycled aggregates. In particular, the provided material, called “Recycled 0–5”, is obtained by recycled concrete and is characterized by a particle size lower than 5.6 mm. The company provides the RA features shown in Table 2, according to UNI EN 126020:2002 + A1:2008 [51].

### 2.2. Methods

#### 2.2.1. Sieving Process

**Figure 1:**
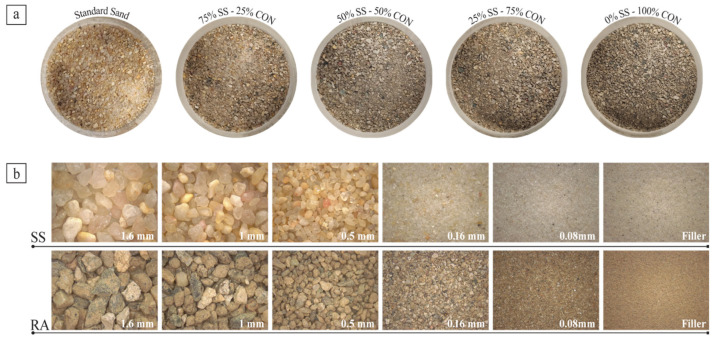
SS and SS-CON mixes (**a**) and comparison between standard sand (SS) and recycled aggregate (RA) particles (**b**).

In order to substitute RS to SS without modifying the particle size distribution, the SS was initially separated into six size fractions through sieving (<0.08, 0.08/0.16, 0.16/0.50, 0.50/1.00, 1.00/1.60, 1.60/2.00 mm). The recycled sand was also screened into the same six particle size fractions. The fractions were successfully blended to obtain four sand samples, called CON, with corresponding fractions of RS in specific ratios to match the particle size distribution of the original SS. The sand mixes (Figure 1a) were created at four different replacement ratios (25%, 50%, 75%, and 100%). The composition of the new sand blends is shown in Table 3. A comparison between the different size fractions of SS and RS is shown in Figure 1b.

#### 2.2.2. Recycled Sand Characterization

The recycled sand (RS) was analyzed by using pycnometry, thermogravimetric analysis (TGA), Field Emission Scanning Electron Microscopy (FESEM), and X-ray Diffraction and X-ray Fluorescence (XRD and XRF, respectively). To analyze the RS density through pycnometry, an Anton Paar Ultrapyc 5000 (Anton Paar Italia S.r.l, Rivoli, Italy) was used. Three SS and RA-CON samples were tested, using an approximate weight of 10 g. The TGA was conducted using a Mettler Toledo 1600 (Mettler Toledo, Milan, Italy), in air atmosphere up to 1000 °C, with a heating ramp of 10 °C/min. The FESEM observations were performed with a Phenom ProX instrument (Thermo Fisher Scientific, Eindhoven, The Netherland), both to assess the crystallization effects occurring in the RS, and to evaluate their impact on the mortar. It provides information about the particle size and distribution within the cement matrix, also enabling the analysis of crack development and fracture patterns. The samples were platinum sputtered for 30 s before FESEM observations (Quorum Sputter Coater, model Q150T S, Laughton, East Sussex, UK). Lastly, the XRD patterns were recorded with a Pan’Analytical X’Pert Pro diffractometer (Malvern Pan’analytical, Worcestershire, UK) equipped with a copper anticathode (λCuKα_1_ = 0.15406 nm) and a linear detector, between 5° and 70° in 2θ, with a step width of 0.026°. The retained fraction at 0.16 mm and the filler fraction of the RS were selected for XRD analysis. Additionally, the chemical composition of the RA was assessed by X-ray Fluorescence using a Rigaku ZSX 100E (Rigaku Holdings Corporation, Tokyo, Japan) instrument through the filler fraction.

#### 2.2.3. Mortar Samples Preparation

For each ratio between RS and total sand, 4 series of mortar samples were prepared, tested and analyzed (named CON, CON-X, CON-Y and CON-Z). For each series, at least three samples were realized; in addition, three reference samples were made for each series.

Making mortar samples followed the mix design outlined in Table 4, using a water-to-cement ratio of 0.5 and a cement-to-aggregate ratio of 1:3 following the EN 196-1 standard [52]. The procedure involved mixing water and superplasticizer with cement in a bowl with an automatic mortar mixer at low speed for 30 s, then gradually adding sand for the next 30 s while switching to high speed for another 30 s. The mixer was then stopped for 90 s, and any mortar on the bowl’s walls was scraped off and added to the mixture. After the break, the mixing was resumed for 60 s at high speed. The first half of the mixture was carefully transferred into steel molds allowing the preparation of three 40 × 40 × 160 mm^3^ prismatic specimens and compacted with 60 jolts. The remaining mixture was then poured into the half-filled molds and compacted with another 60 jolts. The molds were then placed in a room with relative humidity of 100% for 24 h. The samples were finally demolded and placed in a water tank for curing at 24 ± 1 °C for 7 or 28 days. Once the curing time was finished, the samples underwent three-point bending and compression tests to evaluate the mechanical performance following the EN 196-1 standard.

The different CON-X, CON-Y and CON-Z series differ by the way the AD crystallizing agent was added. On the one hand, in the CON-X series, 1 wt% of AD with respect to cement was added at the end of the mixing procedure (as per the recommendation of the producer). Then, the mortar mix was mixed for another 60 s to obtain a homogenous paste. In the case of CON-Y and CON-Z, on the other hand, the AD additive was used to pretreat the sand before preparing the mortar. This approach was used to limit as much as possible the greater absorption of water due to the RS porosity. Thus, in the case of CON-Y and CON-Z, the SS+RS mixture was pretreated with a mix of water and AD (1% AD by weight of sand). Water was added to AD since 1% of the crystallizing agent (13.5 g) would not be always sufficient to reach the total saturation of the RS pores. The water used to pretreat the sand was subtracted from the quantity of water needed in the mortar. The batches were then cured in sealed bags to prevent humidity losses for 15 (CON-Y series) and 45 days (CON-Z series); the mix design for the Y and Z series is also reported in Table 4.

Additionally, for each series, three cylindric specimens of 50 mm in height and 95 mm in diameter, and three cubic specimens of 100 mm side were prepared to perform the rapid chloride permeability test and the water penetration resistance test in accordance with ASTM C1202 [53] and UNI EN 12390-8 [54], respectively, with the aim to evaluate the durability of the specimens.

For all the mixes, it was decided to maintain an almost constant workability; consequently, there is variability in the SP dosage to keep the mortar at the OPC standard workability. The slump test results were reported in Table 5 for all the series of the mix design. As shown in Table 4, CON and CON-X series behaved similarly regarding workability, while CON-Y and CON-Z required a higher amount of SP. In all cases, the SP requirement grows with the RS fraction inside the mortar, due to the higher water uptake from the RS porosity with respect to the mortar with only standard sand.

### 2.3. Mortar’s Characterization

#### 2.3.1. Mechanical Behavior

**Figure 2:**
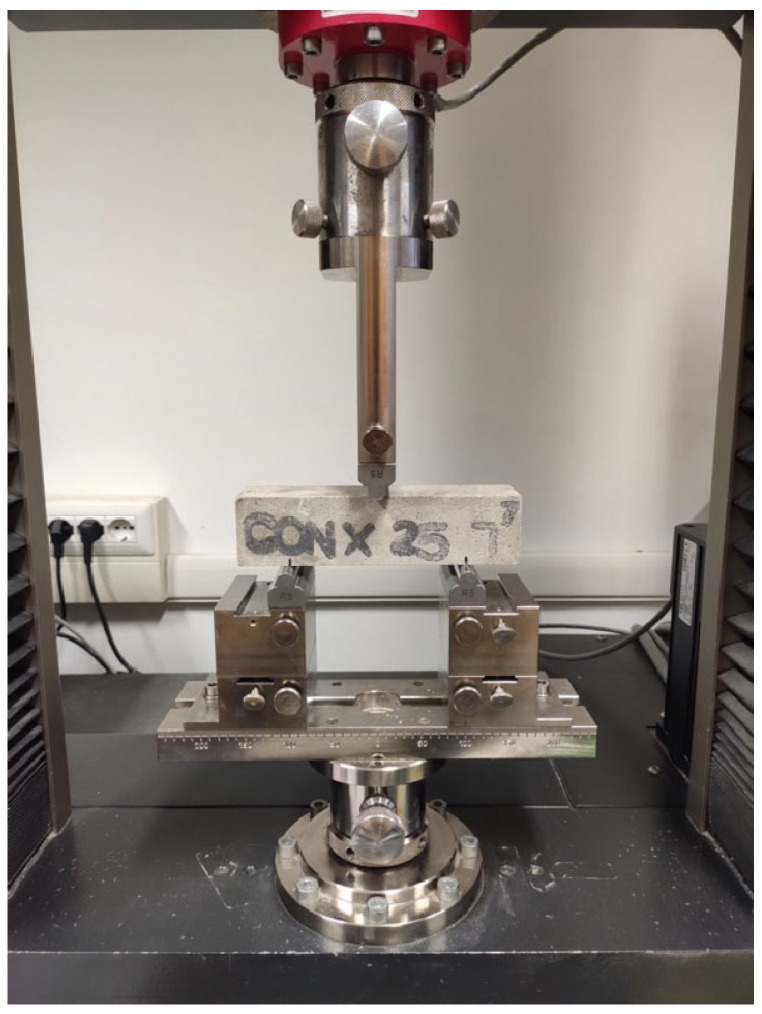
Three-point bending test.

To assess the mechanical behavior of mortar specimens, all the samples underwent a three-point bending (TPB) and compression test following the UNI 196-1 Standard [52]. The TPB test was carried out using a Zwick-Line Z050 single-column machine (ZwickRoell, Ulm, Germany) with a cell load capacity of 50 kN, a pre-load of 5 N, a span of 100 mm, and a testing rate of 50 N/s (Figure 2). The flexural strength was determined using Equation (1):

(1) $$\sigma_{f}=\frac{3F_{max}L}{2bh^{2}}$$

where *F_max_* is the maximum applied force on the prism at the instant of failure, *L* is the effective span, *b* is the prism width and *h* is the height of the specimen under the point of the application of the load.

**Figure 3:**
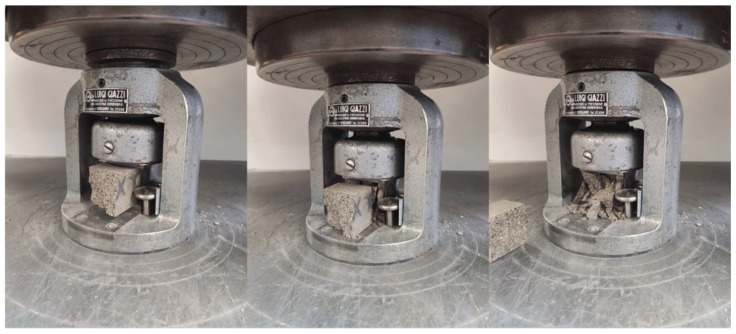
Compressive test of mortar specimens.

After flexural testing, the prisms’ broken portions were subjected to compression testing using a Zwick–Baldwin single-column machine with a load cell capacity of 500 kN and a test velocity rate of 2400 N/s (Figure 3). The compressive strength was calculated by dividing the maximum load by the original cross-sectional area of the specimen:

(2) $$\sigma_{c,max}=\frac{F_{max}}{bh}$$

#### 2.3.2. Chloride Permeability Test

**Figure 4:**
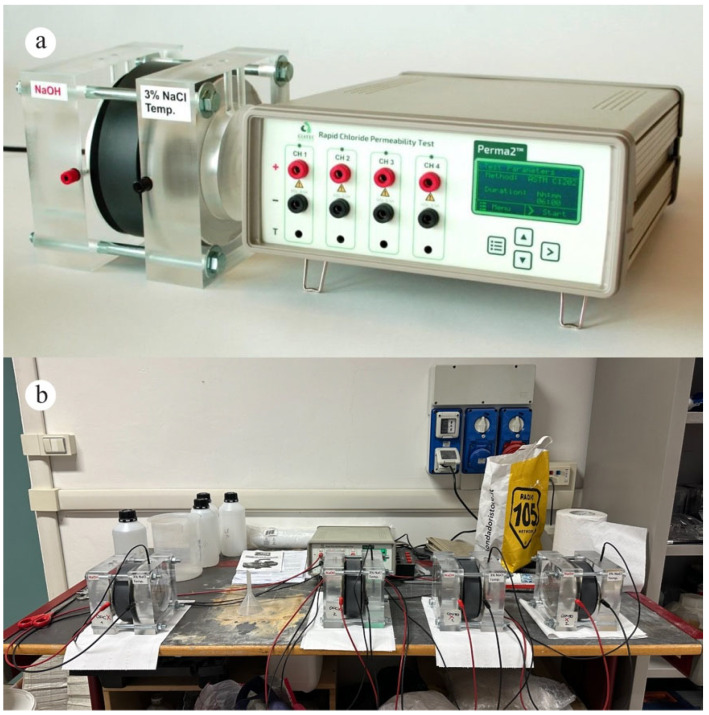
(**a**) Test cell and Perma 2TM voltage applicator. (**b**) Chloride permeability running test.

The chloride permeability test was used to evaluate the ability of mortar samples to resist the penetration of chloride ions, which can cause corrosion of reinforcing steel in structures [53]. For this work, the ASTM C1202 standard was followed. The underlying concept of ASTM C1202 is that anions, when subjected to an electric field, will migrate from the negative electrode to the positive one. When comparing various specimens under identical experimental conditions, the extent of ions’ migration directly correlates with the magnitude of the electrical flow observed. This relationship serves as an indicator of the relative permeability of the tested samples. For the test, 28-day cured samples were used. First, the specimens were allowed to air dry for at least 1 h. Next, a rapid setting coating was applied onto the side surface of each cylindrical specimen and placed on a suitable support to cure as per the manufacturer’s instructions. Once the coating was no longer sticky to the touch (approximately 2 h later), the specimens were positioned inside a vacuum desiccator under an absolute pressure of less than 6650 Pa for 3 h. De-aerated water, previously prepared, was then added to the chamber with the vacuum pump still running until all specimens were completely covered. The vacuum process continued for an additional hour. Following that, the samples were left to soak in water for 18 h. Later, the specimens were carefully positioned inside the designated test cells, as illustrated in Figure 4. The test cells are composed of two containers into which 3% NaCl and 0.3 N NaOH solutions were added. The mortar specimens were then securely held in place between the two containers with the aid of vulcanized rubber gaskets. Then, the leads were connected to a Perma 2 TM voltage applicator (Figure 4a). Finally, the testing phase started and continued for 6 h (Figure 4b). The air temperature surrounding the specimens was maintained within the 20 °C to 25 °C range throughout the test.

#### 2.3.3. Water Penetration Resistance

**Figure 5:**
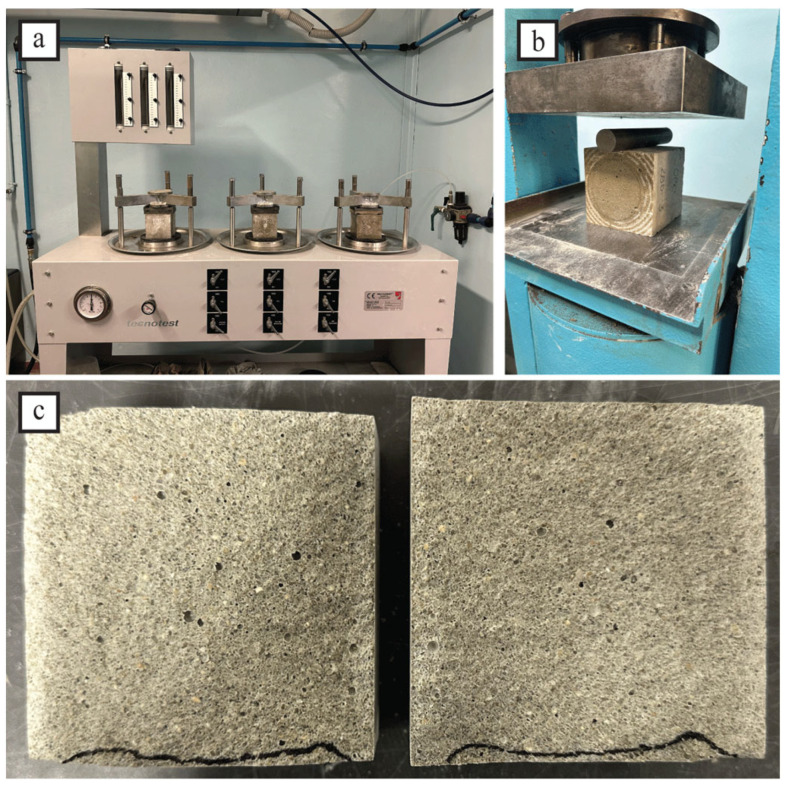
Example procedure for water penetration testing. (**a**) Testing machine for assessing water permeation resistance. (**b**) Sample splitting. (**c**) Determination of water penetration depth.

In order to estimate the porosity of the mortar, the water penetration resistance was evaluated; it is an important property, particularly in areas with high rainfall, high humidity, or exposure to seawater [10]. The test on hardened cement-based materials for determining the penetration depth of water under pressure is regulated by the UNI EN 12390-8 standard [54]. First, three cubic specimens of 100 mm in side, matured for 28 days, were put in a Tecnotest A1 315 test machine (Tecnotest, Modena, Italy) to apply water at a pressure of 500 kPa for 72 h (Figure 5a). After that, each specimen was split in half, perpendicular to the face to which water pressure was applied (Figure 5b). Finally, water penetration marks were drawn immediately after splitting (Figure 5c). The test result is the maximum penetration depth expressed to the nearest millimeter. Notwithstanding, the standard lacks particular indications for interpreting the test results. In this regard, the prescription of the German standard DIN 1045 points 6.5.7.2 and 6.5.7.5 were followed: waterproof concrete presents a water penetration lower than 50 mm, while concrete with a high resistance to chemical attack displays a water penetration lower than 30 mm [55].

## 3. Results

### 3.1. Recycled Sand Characterization

#### 3.1.1. Thermogravimetric Analysis (TGA)

**Figure 6:**
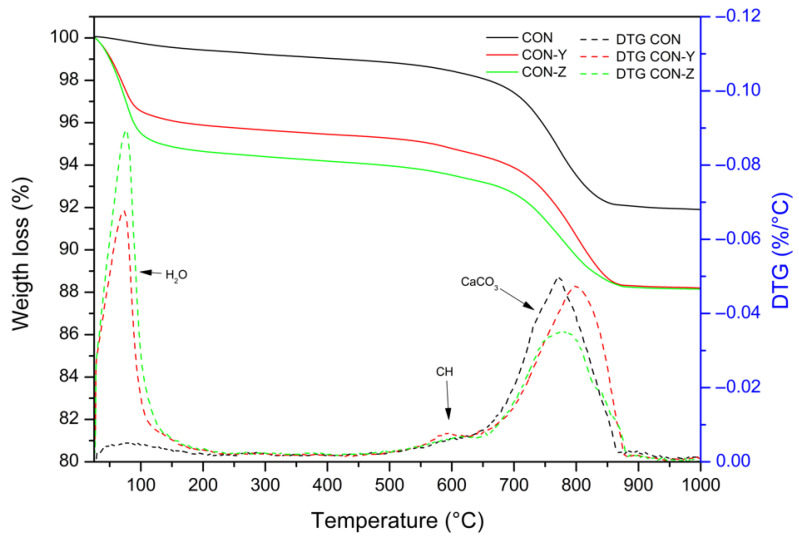
Thermogravimetric analysis (TGA) of CON series.

The TGA was performed on samples of RS untreated and treated with the crystallizing agent (AD), and the results are shown in Figure 6. The weight loss between 25 °C and 100 °C is due to the evaporation of water. This mass loss was more prominent in specimens previously treated with the crystallizing agent (CON-Y and CON-Z), since probably during the pretreatment step water was trapped in the porous structure of the RA. Subsequently, from 100 °C to approximately 600 °C, a slow dehydration process of the hydrated phases of the cement occurred. Around 600 °C, a weak signal due to the decomposition of portlandite (Ca(OH)_2_) was observed, transforming it into calcium oxide (CaO). Furthermore, a significant drop in mass between 700 °C and 870 °C indicated the thermal decomposition of calcium carbonate (CaCO_3_), which was present in the aggregate and resulted from the chemical reaction between calcium hydroxide and carbon dioxide from air. This process led to the formation of calcium oxide and the release of CO_2_.

#### 3.1.2. Field Emission Scanning Electron Microscopy (FESEM) Observations

**Figure 7:**
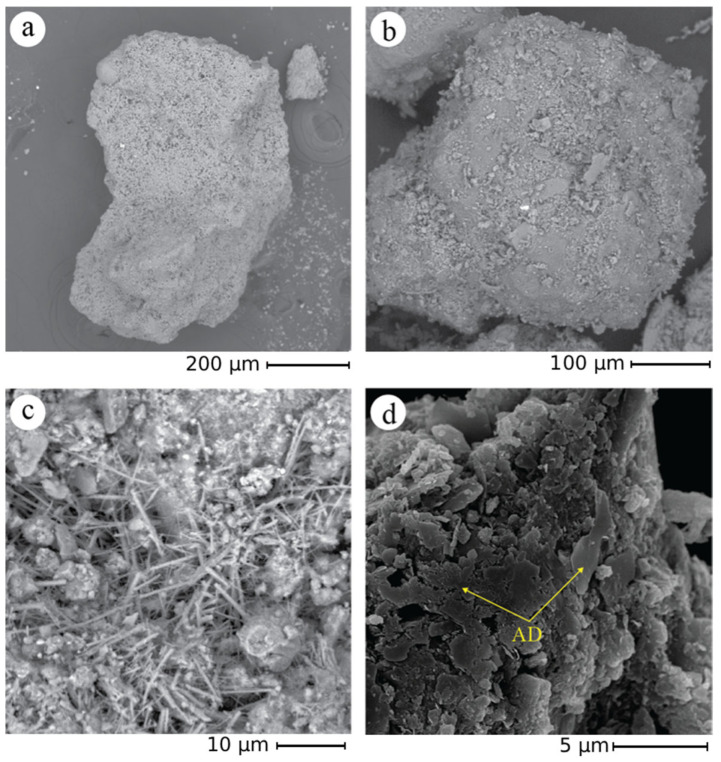
CON particles—FESEM analysis. (**a**) reveals extensive porosity spreads across the particle’s surface; (**b**) showcases the presence of finer particles intricately layered on the surface; (**c**) captures the formation of distinct ettringite structures; (**d**) highlights the formation of AD crystals resulting from the application of the crystallizing agent.

FESEM observations were conducted on the recycled sand used for the CON and CON-Z samples to examine its morphological features, ascertain its capacity for optimal bonding with the cement matrix, and analyze the AD effect (Figure 7). FESEM observations were performed in the retained fractions at 0.16 and 1.00 mm.

In general, the particles exhibit a mixture of sharper edged and angular fractions, as expected for milled aggregates, which have the potential to facilitate enhanced adhesion with the cement matrix. A small fraction of the sand is instead characterized by a rounded shape similar to standardized sand grains. Furthermore, a very fine powder is observed on the particle surfaces, that could have an impact on the mortar preparation process (Figure 7b). In fact, this fine fraction could contribute to the higher water demand observed in mixes incorporating recycled sand. Furthermore, Figure 7c depicts the presence of ettringite. Similarly, in Figure 7d, the formation of AD crystals is readily visible.

#### 3.1.3. X-Ray Diffraction and X-Ray Fluorescence (XRD and XRF)

**Figure 8:**
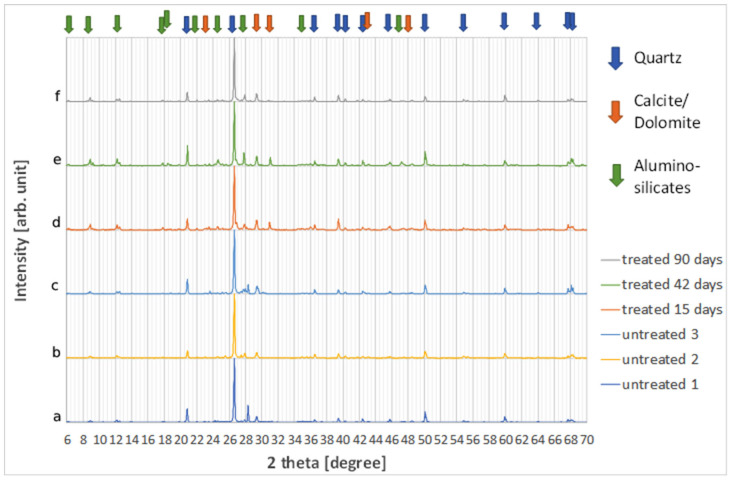
XRD pattern of CON samples before and after treatment with AD. (a–c) untreated RS; (d–f) RS samples after treatment with AD for 15, 45, and 90 days, respectively.

XRD analysis of the recycled sand is depicted in Figure 8. The first three curves, (a), (b), and (c), present the XRD pattern of RA sand without treatments, in order to have an estimation of the homogeneity of this material. The XRD patterns were rather similar, revealing the presence of quartz and calcite as major constituents. However, several minor compounds are also present, in different amounts in the different samples, belonging to different mineral families. Clinochlores (magnesium iron aluminosilicates), phyllosilicates (aluminum and magnesium silicates), feldspars (alumino-silicates), dolomite (calcium magnesium carbonate) were observed. The identification of some of these minority phases is uncertain since the corresponding peaks are very low. Moreover, it must be stressed that X-ray diffraction cannot identify amorphous or poorly crystalline phases. Finally, gypsum was never found in the samples investigated. Most of these phases, i.e., clinochlores, come from the aggregates fraction, while calcite could have different origins: from aggregates, as a cement filler, and from the concrete carbonation process. Curves (d), (e), and (f) show instead the samples after treatment with AD for 15, 45, and 90 days, respectively. No significant modification of the XRD spectra can be observed, the slight variations observed in all the tests are probably related to the non-perfect homogeneity of the sand samples.

Table 6 shows the composition of the RA powder from XRF analysis. The high presence of silica, more than 40%, is due to silicates and quartz. Calcium, magnesium, aluminum, and iron are consistent with the typical cement and aggregate composition. The presence of quartz and calcite is also confirmed by the XRD pattern (Figure 8).

### 3.2. Mechanical Behavior

#### 3.2.1. CON Series

**Figure 9:**
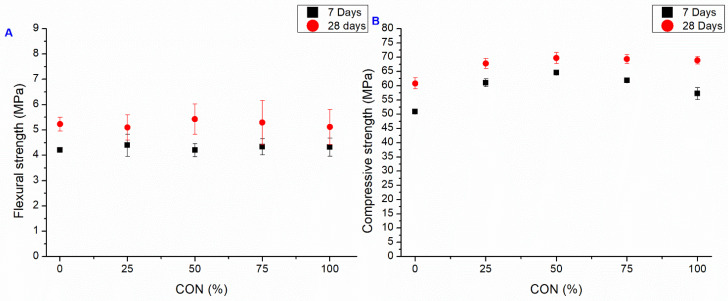
Flexural (**A**) and compressive strength. (**B**) Average value at 7 and 28 days of CON series (bars represent the standard deviation value of each series).

The three-point bending and compression test results for the CON series, i.e., the case where RAs replace SS without any addition of AD, are depicted in Figure 9. As previously reported, using RAs in mortars necessitated a higher water content during the mixing phase than SS due to RA’s intrinsic porosity. To overcome this challenge, an SP agent was used for all specimens where SS was replaced, allowing the same workability as the standard mortar and easy casting of all the mixes. The SP acts as a lubricant, reducing internal friction within the mortar matrix. This reduction in friction allows for better particle packing and increases interparticle contact, resulting in enhanced load transfer and improved overall strength [56]. However, mixes with 75% and 100% SS replacement rates required very high SP dosages, as reported in Table 4.

The three-point bending results (Figure 9A) present a similar strength than the control mixture, while there is a marked increase in compressive strength (Figure 9B). The observed enhancement in the compressive strength of the specimens made with RAs can be attributed to its inherent characteristics: as a porous aggregate, the RA can absorb free water present in the mixture. This water absorption process reduces the effective water-to-cement ratio and leads to notable and promising improvements in the mechanical performance of the mortars, even at high SS replacement rates. After 7 days, the compressive strength is increased by at least 12.5% for all the samples, and 26.9% for the best one (CON 50), while after 28 days, the increment is at least 11.5% for all the samples, and 14.6% for the CON 50 sample. A high RA fraction seems to slow down the development of compressive strength, but after 28 days the strength is comparable for all the samples.

#### 3.2.2. CON-X Series

**Figure 10:**
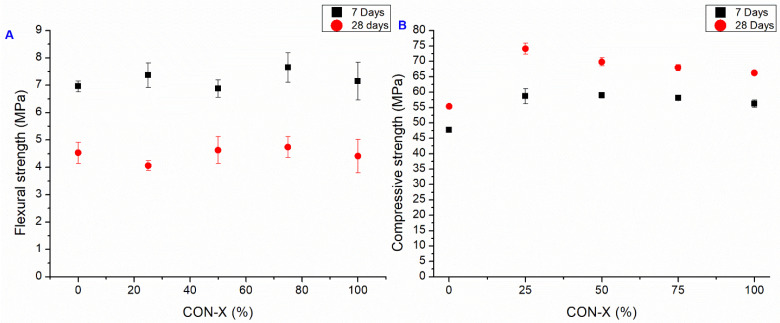
Flexural (**A**) and compressive strength (**B**). Average value at 7 and 28 days of CON-X series (bars represent the standard deviation value of each series).

**Figure 11:**
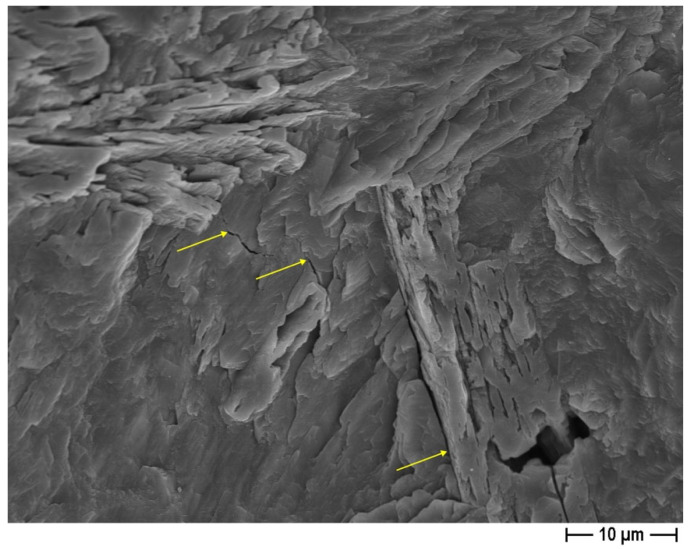
AD crystal growth in pores in the standard mortar (OPCX) at 28 days (FESEM × 2k; arrows indicate microcracks probably due to AD).

In the second mortar series, CON-X, the crystallizing agent was incorporated at the end of the mixing process. The mechanical testing results of this series are presented in Figure 10, showcasing the notable impact of AD on the early-stage flexural strength capacity (observed after 7 days). However, after 28 days, a significant decrease in this property is observed compared to the 7-day specimens, bringing it back to a value similar to the CON series. In fact, as the composition of the crystallizing agent is unknown, only hypothesis can be proposed to explain the trend of the observed mechanical results. Usually, crystalline admixtures contain Portland cement, silica sand (reactive silica), and other reagents (reactive components, like sodium silicate, for example) [57]. This chemical admixture then reacts with Ca(OH)_2_ to produce insoluble crystals such as calcium silicate hydrates (C–S–H). However, the influence of AD on the mechanical strength of the cementitious matrix is rather clear: in the CON series (without the use of AD), the flexural strength increases with the curing time, whatever the untreated recycled aggregates content, while in the CON-X series, with the crystallizing agent added to the cementitious matrix, the 28-day flexural strength is lower than the 7-day one. As the portlandite content in the cementitious matrix increases over time during the curing step, one can suppose that the reaction of the crystallizing agent is not completed after 7 days and will proceed in the hardened cementitious matrix, causing some possible microcracks. This interpretation is supported by the observation of these microcracks by FESEM (Figure 11, yellow arrows). Both after 7 and 28 days, the RA to SS substitution ratio seemed to have no significant effect on flexural strength, as also observed in the case of the CON samples.

Regarding compressive strength, the presence of the AD agent did not cause the same improvement at 7 days, as it did on the flexural strength. Instead, notable improvements with respect to OPC-X were observed across all cases. The best case was CON-X 25, with improvements of 23% and 33.9% at 7 and 28 days, respectively. However, no significant improvement is observed with respect to the CON samples, on the contrary a small decrease is generally observed with respect to the samples without AD at 7 days, while at 28 days the strength is similar to the CON case, and higher in the case of CON-X 25.

#### 3.2.3. CON-Y Series

**Figure 12:**
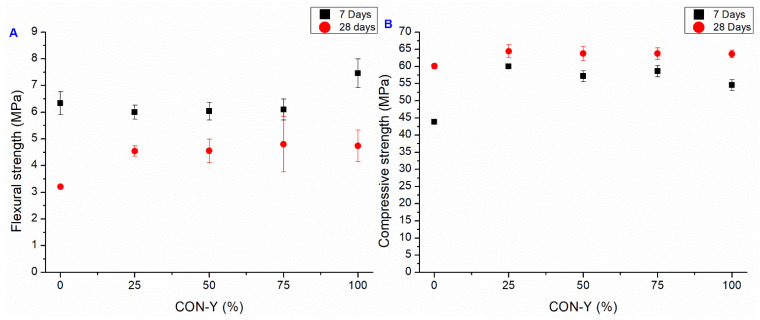
Flexural (**A**) and compressive strength (**B**). Average value at 7 and 28 days of CON-Y series (bars represent the standard deviation value of each series).

The Y series addresses the issues encountered with the mortars in the CON-X series, wherein an alternative approach involving the direct utilization of AD in the aggregate was employed. According to the producer, AD activation occurs upon contact with the cement hydration compounds that are present in the recycled aggregate, prompting a decision to subject the treated sand to a 15-day curing period to allow for the reaction to happen, and fill in the RA pores, before the preparation of mortar. The flexural and compressive strength results of the CON-Y samples are represented in Figure 12.

Regarding flexural strength, it is evident that the OPC-Y and CON-Y cases exhibit a similar pattern, wherein a significant increase in strength at 7 days is observed, while the strength is reduced after 28 days. The same hypothesis proposed for CON-X series can be proposed also in this case: the chemical reactions of the crystallizing agent were not completed after 15 days and led to local microcracking. This effect, however, is smaller than in the OPC-X and CON-X cases. At 28 days, the CON-Y samples had similar flexural strength to CON-X, but smaller than in the CON series.

Regarding compression strength, the improvement of strength of the CON-Y samples with respect to OPC-Y is still observed, but the strength of the samples where the RA substituted for SS is smaller than in the CON case, both at 7 days and at 28 days.

#### 3.2.4. CON-Z Series

Since the crystallization reactions of the AD agent could require a long time to happen, a fourth set of samples, Z-series, was realized with a higher permanence time of AD agent in the RA pores (45 days).

**Figure 13:**
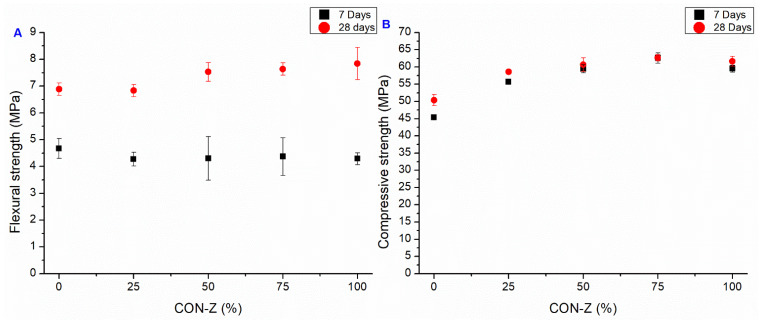
Flexural (**A**) and compressive strength (**B**). Average value at 7 and 28 days of CON-Z series (bars represent the standard deviation value of each series).

**Figure 14:**
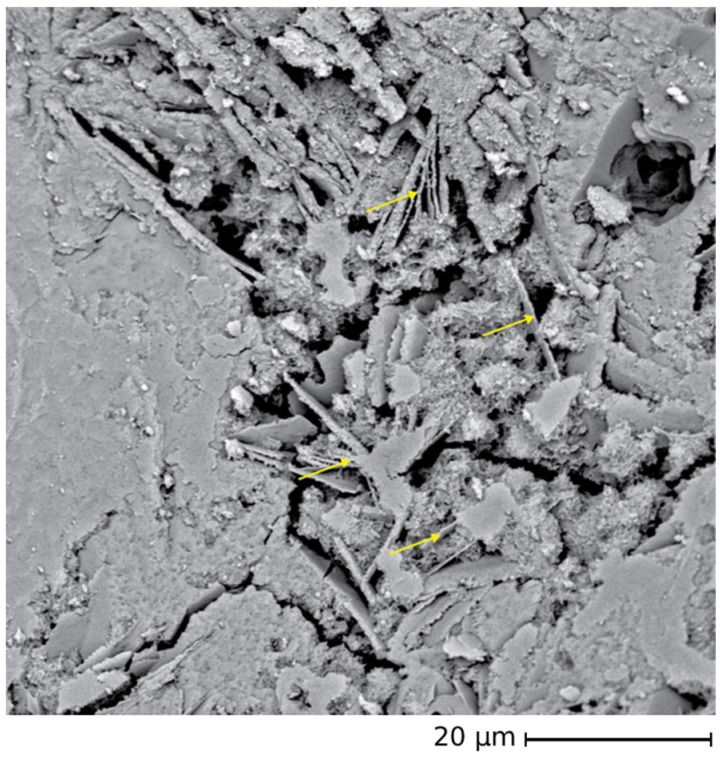
AD crystal growth inside the matrix’s pores (FESEM ×3.5k—OPCZ at 28 days; arrows indicate crystals grown in pores).

**Figure 15:**
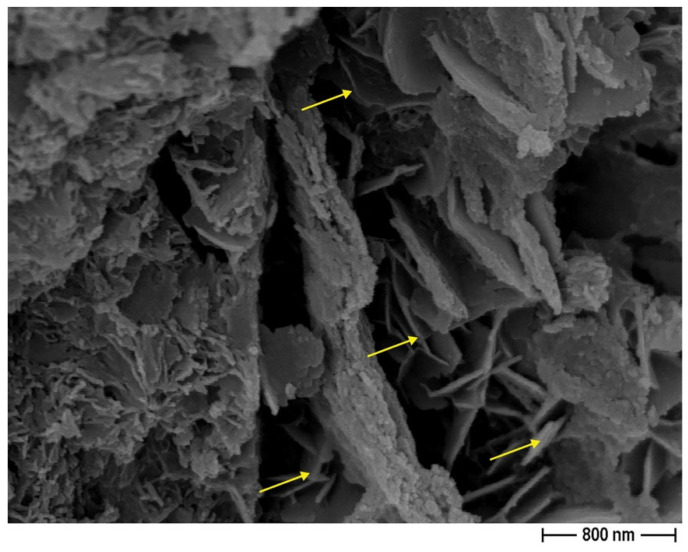
AD crystal growth inside the aggregate’s pores (FESEM ×30k; arrows indicate crystals grown in pores).

The results of mechanical testing of CON-Z series are presented in Figure 13. The significant improvement in flexural strength at 28 days is particularly noteworthy, with an enhancement from 33 to 53% with respect to the CON samples. The flexural strength at 7 days is instead in line with the CON samples. The highest improvement in bending performance compared to the standard mortar was observed in the CON-Z 100 sample. This enhancement could be attributed to the long-term AD crystal growth inside the matrix and aggregate’s porosity, enhancing the material resistance: the chemical reactions can be considered as almost completed and no microcracking can be expected in the cementitious matrix, leading to a 28-day flexural strength higher than the 7-day one. The FESEM analysis corroborates this hypothesis, showing crystal growth inside the matrix and aggregate pores in Figure 14 and Figure 15, respectively (yellow arrows). These results suggest that a longer pretreatment and a longer curing time was needed to observe the AD influence, even if this mechanism did not match perfectly with the one observed for the CON-X and CON-Y samples, where a short-term strength improvement was observed. Regarding compressive performance, the results closely align with the previous series, where the mechanical strength is smaller than in the case of the CON samples. It is worth noting that also in this case all the mortars containing RAs exhibited superior strength compared to the reference. Regarding compressive strength, it can be observed that results at 7 and 28 days are very similar, suggesting an acceleration of strength development. Again, this result is not very consistent with the results on flexural strength.

### 3.3. Chloride Permeability Test

The specimens for the chloride permeability test were chosen on the basis of the mechanical results presented in the previous section. Also, the amount of waste material used, the amount of superplasticizer utilized, and the evaluation of the crystallizing agent’s influence were kept into account. Given these considerations, the samples substituted at 50% with RA were chosen since they presented good mechanical properties and required an acceptable amount of SP. Also, their mechanical properties were not uncommon within their sets. Therefore, the study’s samples included OPC, OPC-X, OPC-Z, CON 50, CON-X 50, and CON-Z 50. The Y-series was excluded in favor of the Z-series.

**Figure 16:**
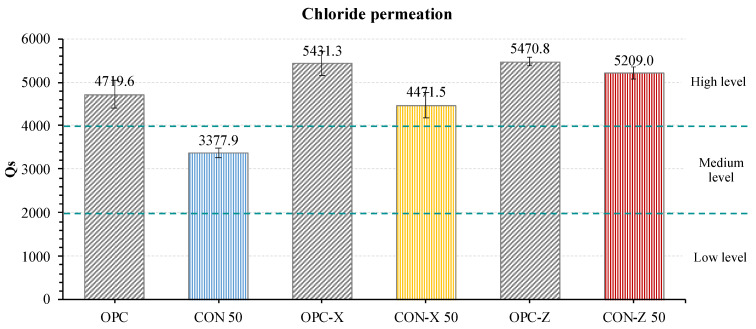
Rapid chloride permeability index results.

The results obtained from the ASTM C1202-19 test are shown in Figure 16. The OPC samples exhibited a “high” rapid chloride permeability index (RCPI), indicating elevated electrical conductivity and chloride ion permeability. Very interestingly, CON 50 specimens, including 50% RA, showed a lower RCPI, i.e., a reduced electrical conductivity and chloride ion permeability compared to the standard mortar. This result is probably due to the presence of recycled aggregates, which reduce the water-to-cement ratio, allowing the formation of a more compact cement matrix and thus a less porous material.

Conversely, all the samples treated with AD (both X and Z series) displayed an RCPI higher than OPC, indicating increased electrical conductivity and chloride ion permeability. This would suggest that the AD treatment did not significantly improve the chloride ion permeability. However, Liang and Ji [58] proposed that when the specimens are submerged in water before the test, any surplus free alkaline components persist in dissolving. Since AD contains alkaline elements (mainly sodium), it is probable that the addition of AD brings to the presence of additional ions in the system that increase the electrical conductivity of the solution and thus give a false result for the RCPI. In practice, it is probably incorrect to compare OPC with the OPC-X or OPC-Z samples. Furthermore, the observed results contrast the findings reported by Yang et al. [59], who assert that incorporating a crystallizing agent improves chloride resistance performance. However, the authors did not adhere to the ASTM standard followed in this work.

In any case, an observation can be conducted regarding the comparison between samples with and without recycled aggregates; in all the cases, samples containing RAs present a lower RCPI than samples with only SS, suggesting a beneficial effect of the introduction of recycled aggregates, due to abovementioned effect of the reduction in the effective water-to-cement ratio.

### 3.4. Water Penetration Resistance

**Figure 17:**
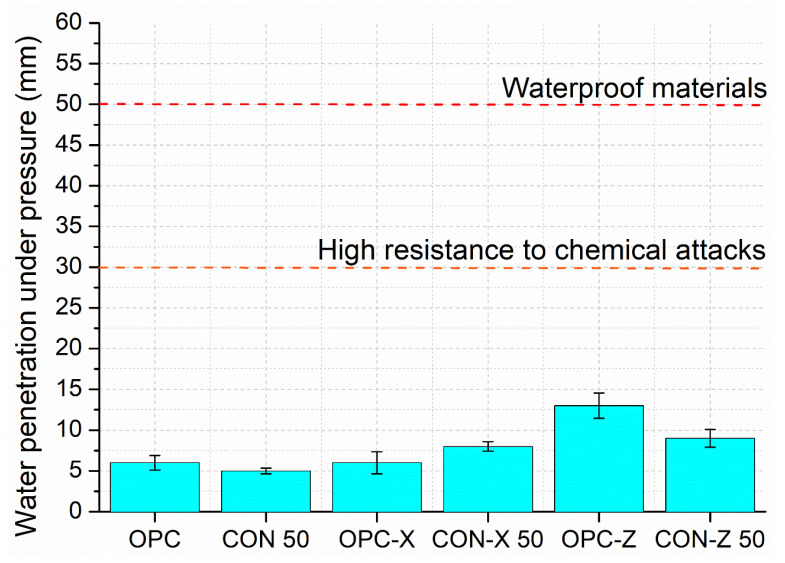
Water penetration resistance results.

The depths of water penetration under pressure results obtained following the UNI EN 12390-8 standard are depicted in Figure 17. The test was again performed on OPC, OPC-X, OPC-Z, CON 50, CON-X 50, and CON-Z 50 samples. A lower depth of water penetration generally indicates improved water resistance and reduced permeability of the mortar. The OPC and CON 50 samples exhibited the lowest penetration depths of 6 and 5 mm, respectively. The X-series samples showed penetration depths of 6 and 8 mm for OPC-X and CON-X 50 specimens, respectively. The OPC-Z and CON-Z 50 samples showed the highest penetration depths of 13 mm and 9 mm, respectively.

**Figure 18:**
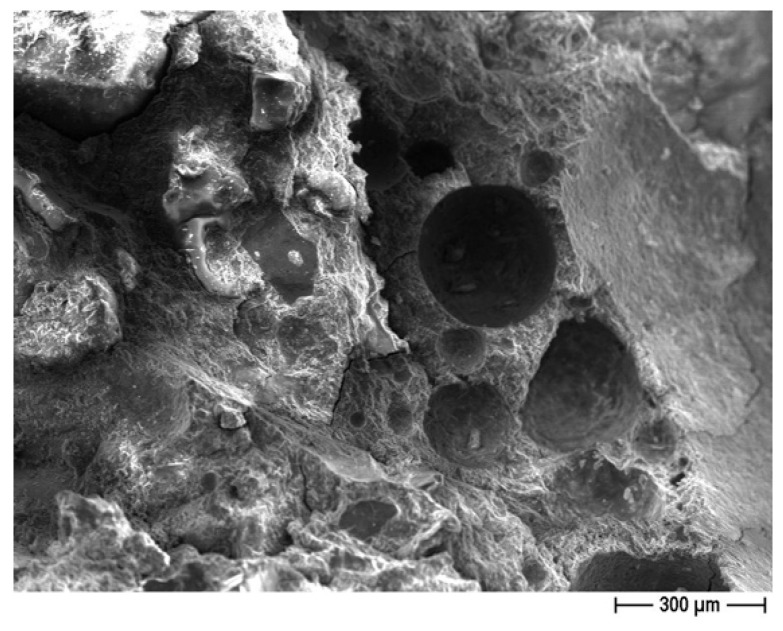
OPC sample without any treatment (FESEM ×80).

**Figure 19:**
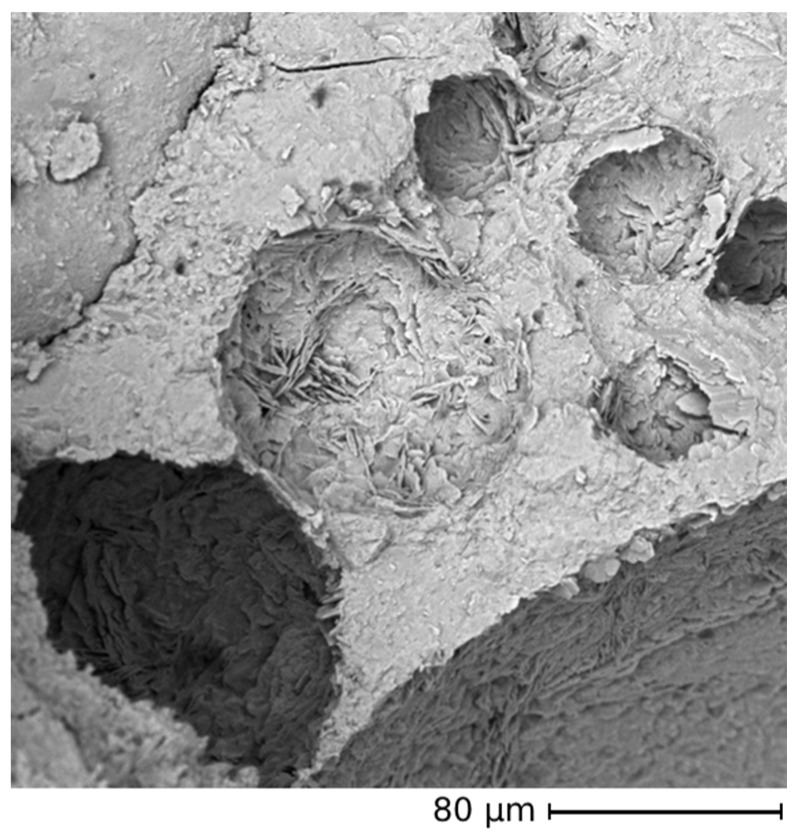
Pore sealing detail (FESEM ×1k—OPCZ at 28 days).

It is interesting to compare the mechanical results of OPC, OPC-X, and OPC-Z with the water penetration results, since the reduction in compressive strength corresponds to an increased water penetration, as expected. The samples OPC and OPC-X are both made with standard sand, but the latter containing the crystallizing agent added to the cement, showed the same value of water penetration depth. In this case, the cementitious matrix is probably already compact, and the crystallizing agent does not make any contribution. In the case of the OPC-Z samples, while standard sand was still used, the AD content was higher and could explain the difference in the water penetration depth with respect to the two previous samples, considering that the crystallizing agent should react with portlandite which is not present in standard sand. This could lead to a weaker interface with the cementitious paste. However, in the case of OPC-X, cracks were observed at 28 days causing a reduction in flexural strength, while in the case of OPC-Z, flexural strength increased. Nevertheless, FESEM images show that the crystallizing agent initiates a pore-filling process (Figure 18 and Figure 19).

In the case of the CON, CON-X, and CON-Z samples, one can again consider mechanical strength, which is similar for CON 50 and CON-X 50 and lower for the CON-Z 50 samples. The CON-X 50 and CON-Z 50 samples showed close water penetration depths, only slightly higher than the OPC and OPC-X samples. The possible cracks in CON-X 50 probably have a limited extension and make only a slight contribution to water penetration. More surprising is the water penetration depth of the sample CON 50, which showed the lowest value while containing the same content of recycled aggregates and of superplasticizer as the sample CON-X 50. This could be since the crystallizing agent reacts with portlandite; in systems with a high recycled fines content, the availability/redistribution of Ca(OH)_2_ in the interfacial transition zone may be unfavorable, leading to a weaker or more porous transition zone [60].

This partially justifies the trend of the values of water penetration, even if also in this case the AD agent seems to have a negative effect.

Since the UNI EN 12390-8 standard does not provide specific guidelines for interpreting the test results, the German standard DIN 1045 was followed, specifically in points 6.5.7.2 and 6.5.7.5. According to the German standard, a water penetration depth of less than 50 mm indicates a material with waterproof properties, suggesting excellent water resistance. Furthermore, a water penetration depth of less than 30 mm indicates a high resistance to chemical attacks, implying enhanced durability in aggressive environments. All the tested samples presented water penetration values much lower than 30 mm.

Table 7 compares the results obtained in this work with those from the literature published in the last 18 months. Only the methods describing the strengthening of the adhered mortar on recycled aggregates were considered, for the sake of comparison with the method proposed in this paper. Thus, all the works including a mechanical/thermal grinding step aimed at removing the old cement paste were voluntarily omitted. Finally, articles dealing with fine, coarse, or both kinds of aggregates were reported, because the proposed solution can be applied to all aggregates, independently of their size. Even when taking into consideration some limitations, as mentioned above, a high number of papers were found, highlighting the interest of this topic in the scientific literature.

## 4. Conclusions

In recent decades, effective management of construction and demolition waste (CDW) has emerged as a significant challenge for the construction industry, which is increasingly recognizing the imperative to safeguard the natural environment and optimize the utilization of natural resources. Furthermore, with the adoption of new building materials, there is a pressing need for these materials to match or exceed the performance standards of existing ones, both structurally and in terms of sustainability.

This study advances the understanding of recycled aggregates (RAs) derived from waste concrete and explores their potential application in mortar formulations, focusing on evaluating their mechanical, physical, and durability properties. Untreated and treated sand, modified with a crystallizing agent to enhance its performance, were investigated. Physical characterization of RAs through techniques such as thermogravimetric analysis (TGA), X-ray Diffraction (XRD), X-ray Fluorescence (XRF), and Field Emission Scanning Electron Microscopy (FESEM) indicated similarities between untreated and treated recycled sand, both predominantly composed of quartz and calcite. Additionally, the optimal dosage of a superplasticizer was examined.

Mechanical testing revealed that substituting standard sand with fine recycled aggregates while maintaining consistent workability significantly impacted compressive strength. In fact, in most of the cases the strength of samples containing RAs was higher than samples with only SS. Particularly interesting the 25% and 50% substitutions with RA, where the strength improved up to 15% with respect to a standard mortar, and the superplasticizer dosage did not exceed standard values. Mortars utilizing recycled sand treated with crystallizing agent AD imposed higher superplasticizer dosages to maintain equivalent workability to standard mortar, reducing their interest in application. Furthermore, the addition of a crystallizing agent failed to improve strength over the samples where this agent was not employed. However, some interesting effects were observed both in flexural strength and in compressive strength when the crystallizing agent was added. First, in the series X and Y (AD added during the preparation of the mortar paste, or RAs saturated with AD agent for 15 days prior to mortar paste preparation, respectively) the 7-day flexural strength improved significantly, while in series Z (RA saturated with AD agent for 45 days prior to mortar paste preparation) the flexural strength increased at 28 days. Second, compressive strength in series Z seems to develop faster, even if the final strength is smaller than in the case of mortar without AD agent.

Finally, the mortar durability was not positively affected by the addition of AD agent, as evidenced by inferior performance in chloride permeability and water penetration resistance tests.

Overall, the study presents promising findings, demonstrating the potential to achieve comparable or enhanced mechanical properties to standard mortar through substantial sand replacement with recycled concrete aggregates modified by a simple process involving commercial products. It underscores the feasibility of integrating sustainable practices into the construction sector, aligning with the principles of a circular economy. Finally, this research contributes to advancing clean production by advocating for the efficient utilization of natural resources. Further work will explore the modification of coarse aggregates and their incorporation in concrete in combination with modified RFAs.

## Figures and Tables

**Table 1:** CEN standard sand, retained% and retained mass.

Square Mesh Size [mm]	Cumulative Retained [%]	Retained [%]	Retained Mass [g]
2.00	0.0	0.0	0.0
1.60	7.0	7.0	94.5
1.00	33.0	26.0	351.0
0.50	67.0	34.0	459.0
0.16	87.0	20.0	270.0
0.08	99.0	12.0	162.0
Filler	100.0	1.0	13.5

**Table 2:** Recycled 0–5.6 by F.G. S.r.l.

Density of Particles [g/cm^3^]	Purity		Water Absorption [%]
	Powders Content	Sand Equivalent [%]	
2.46	f22	48	4.73

**Table 3:** Sand mixes’ compositions.

Size [mm]	CON 25%		CON 50%		CON 75%		CON 100%	
	SS [g]	RA [g]	SS [g]	RA [g]	SS [g]	RA [g]	SS [g]	RA [g]
1.6	70.90	23.60	47.30	47.30	23.60	70.90	0.00	94.50
1.0	263.30	87.80	175.50	175.50	87.80	263.30	0.00	351.00
0.5	344.30	114.80	229.50	229.50	114.80	344.30	0.00	459.00
0.16	202.50	67.50	135.00	135.00	67.50	202.50	0.00	270.00
0.08	121.50	40.50	81.00	81.00	40.50	121.50	0.00	162.00
Filler	10.10	3.40	6.75	6.75	3.40	10.10	0.00	13.50
Total		1350.00		1350.00		1350.00		1350.00

**Table 4:** Mix design for each series.

Series	Specimen ID	W/C	Cement [g]	Water [g]	Sand		SP [%]	AD [g]
					SS [g]	RS [g]		
1	OPC	0.50	450.00	225.00	1350.00		0.25	-
	CON 25	0.50	450.00	225.00	1012.50	337.50	1.25	-
	CON 50	0.50	450.00	225.00	675.00	675.00	1.70	-
	CON 75	0.50	450.00	225.00	337.50	1012.50	2.80	-
	CON 100	0.50	450.00	225.00	-	1350.00	5.00	-
								
2	OPC-X	0.50	450.00	225.00	1350.00	-	0.25	4.50
	CON-X 25	0.50	450.00	225.00	1012.50	337.50	1.25	4.50
	CON-X 50	0.50	450.00	225.00	675.00	675.00	1.70	4.50
	CON-X 75	0.50	450.00	225.00	337.50	1012.50	2.80	4.50
	CON-X 100	0.50	450.00	225.00	-	1350.00	5.00	4.50
3&4								
	OPC-Y/Z	0.50	450.00	225.00	1350.00	-	0.10	13.50
	CON-Y/Z 25	0.50	450.00	223.45	1012.50	337.50	2.00	13.50
	CON-Y/Z 50	0.50	450.00	213.40	675.00	675.00	3.10	13.50
	CON-Y/Z 75	0.50	450.00	200.85	337.50	1012.50	4.90	13.50
	CON-Y/Z 100	0.50	450.00	188.30	-	1350.00	7.10	13.50

**Table 5:** Slump test for each series.

Series	Specimen ID	Slump [mm]
1	OPC	36
	CON 25	35
	CON 50	35
	CON 75	36
	CON 100	32
2	OPC-X	43
	CON-X 25	45
	CON-X 50	44
	CON-X 75	44
	CON-X 100	45
3	OPC-Y	42
	CON-Y 25	40
	CON-Y 50	42
	CON-Y 75	40
	CON-Y 100	43
4	OPC-Z	42
	CON-Z 25	40
	CON-Z 50	42
	CON-Z 75	40
	CON-Z 100	43

**Table 6:** XRF pattern of RA samples (in mass percentage).

Component	Mass%
LOI-Flux	12.000
Na_2_O	1.380
MgO	4.750
Al_2_O_3_	9.100
SiO_2_	42.800
P_2_O_5_	0.157
SO_3_	3.420
Cl	0.079
K_2_O	1.730
CaO	19.300
TiO_2_	0.392
Cr_2_O_3_	0.067
MnO	0.169
Fe_2_O_3_	4.490
NiO	0.033
CuO	0.011
ZnO	0.017
As_2_O_3_	0.009
Rb_2_O	0.010
SrO	0.037
Y_2_O_3_	0.002
ZrO_2_	0.035

**Table 7:** Comparison of the proposed solution with the literature results.

Aggregates’ Treatment	Water Absorption or Porosity	Mechanical Strength	Ref.
Enzyme-induced carbonate precipitation (EICP) modified RAs	7.01% water absorption reduction with respect to untreated RA	6.05% increase in concrete’s compressive strength with respect to untreated RAs (50.85 MPa, w:c = 0.39, 100% replacement rate)	[61]
Nano-silica (NS) solution (2 wt%) immersion + carbonation treatment modified RCAs	5.13% water absorption for hydrophobic NS treated and carbonated RAs (1.42% for natural aggregates)	34.9% increase in concrete’s compressive strength with respect to untreated RAs (40.29 MPa, w:c = 0.46, 50% replacement rate)	[62]
Soaked 3% bacterial liquid + 0.1 mol/L calcium acetate and 20% CO_2_ carbonation modified RAs	After 50 freeze–thaw cycles, the mass loss of concrete made with treated aggregates was 0.49%, while that of concrete made with untreated aggregates was 4.99%	32% increase in concrete’s compressive strength with respect to untreated RAs (31.48 MPa, w:c = 0.6, 100% replacement rate)	[63]
*Bacillus cereus* modified coarse aggregates	40% reduction in water absorption for treated coarse aggregates with respect to untreated ones	Same compressive strength resistance as concrete made with NA. 11% higher split tensile strength (39.59 MPa, w:c = 0.44, 40% replacement rate)	[33]
Pozzolan (silica fume, fly ash, nano-silica) slurry impregnation or carbonation of coarse aggregates	3.9–4.2% water absorption for treaded aggregates against 5.3% for untreated ones	Up to 55.2% increase in compressive strength for silica fume treated mortar (w:binder = 0.5 and RCA:binder = 2.5)	[64]
Microbial-induced carbonate precipitation (MICP)-modified fine aggregates	50.47% reduction in water absorption after 4 cycles of modification	Flexural strength of mortar made with modified aggregates increased by 23.3% compared to untreated one (w:c = 0.5)	[65]
NS soaking + carbonation	Sulfate penetration depth reduced by over 30% for modified RAs compared to untreated ones	Compressive strength of NS-modified RAs after 28 days of sulfate attack increased by 15% with respect to unmodified RA	[66]
Calcium carbonate precipitation from urea and calcium acetate upon heating	32% water absorption reduction for soaked RAs kept at 80 °C for 5 days with respect to untreated RA	Compressive strength of concrete decreased by 2.2%, 3.5%, 8.4%, and 10.2% for replacement ratios of 30%, 50%, 70%, and 100%, respectively, compared to control made with NAs	[67]
MICP-modified coarse aggregates	Up to 17.9% reduction in water absorption for treated aggregates with respect to untreated ones	17.7% increase in compressive strength compared to untreated aggregates (w:c = 0.5, 100% coarse aggregates replacement)	[68]
Carbonation + coating with an alkali-activated fly ash–slag slurry	Up to 31.43% reduction in water absorption for aggregates first carbonated under 20% CO_2_ and 70 RH%, then impregnated with a saturated lime solution	58.6% increase in concrete’s compressive strength (45.68 MPa)	[69]
Carbonation of coarse RA	5.4–7.1% water absorption for treated RAs with respect to 6.8–8.4% for untreated RA. Coarser RA (10–20 mm) absorbed less water than smaller RA (5–10 mm).	10.5% increase in compressive strength for concrete made with treated RAs compared to the one made with untreated RA; only ≈80% of compressive strength with respect to concrete made with NAs (w:c = 0.38 for NA, w:c = 0.5 for treated RA, 100% replacement)	[70]
RA pre-soaked in 5% silane emulsion	65.2% water absorption reduction	Increase in average flexural fatigue life by 28.5, 36.9, and 44.2 times at the stress levels of 0.6, 0.7, and 0.8 after 140 freeze–thaw cycles (w:c = 0.5, 100% replacement)	[71]
Pre-impregnation or pre-spraying of RAs with calcium phosphate (CaP) solutions	When CaP to RA was 0.003 g/g, the water absorption of pre-sprayed and pre-impregnated treated RAs was 18.02% and 21.61% lower than that of untreated RA, respectively.	When the ratio of CaP to RA was 0.001 g/g, compressive strength increased by 31.52% with pre-spray treatment (100% replacement). In samples with 50% RA and 50% NA, when the unit weight ratio of CaP to RA was 0.003 g/g, the compressive strength increased by 16.52% with pre-impregnation (w:c = 0.35 in all tests).	[72]
Accelerated carbonation (101 kPa, 99.99%, 24 h) and NS solution (2%) immersion	26.4% reduction in water absorption	n.e.	[73]
Accelerated carbonation (150 kPa, 99.9%, 72 h)	23.3% water absorption reduction for treated aggregates (4.86%) compared to non-treated ones (6.34%) (NA = 0.8%)	14.3% and 22.1% decrease in compressive strength and elastic modulus, respectively, with respect to NAs (w:c = 0.53, 100% replacement of coarse aggregates)	[74]
Immersion in a slurry with 15% fly ash, 3% gypsum, and 22% polyacrylate emulsion for 180 s	Saturated surface–dry water absorption rate decreased from 15.0% to 9.5% for treated aggregates with respect to untreated ones	54.5% increase in compressive strength (w:c = 0.4, 100% replacement of coarse aggregates)	[75]
Vacuum impregnation with a 4% NS slurry	2.81% porosity for treated aggregates compared to 4.2% for untreated aggregates (estimated by X-CT)	18.39% increase in compressive strength compared to non-treated aggregates: 55.97 and 47.3 MPa, respectively (w:c = 0.45, 100% replacement of coarse aggregates; 57.9 MPa for the concrete made with NA)	[76]
Impregnation with a 3% NS solution	36.96% porosity reduction with a decrease of 14.35% of average pore size for treated aggregates with respect to untreated ones	41.68 MPa and 34.56 MPa, 52.35 MPa and 44.16 MPa, and 63.22 MPa and 54.25 MPa for treated and untreated aggregates (100% replacement of coarse aggregates) for w:c = 0.51, 0.41, and 0.36, respectively	[77]
Soaking in cement fly ash slurries (30% and 70% concentration)	5.4% water absorption for 24 h soaked aggregates in a 70% concentrated solution compared to 8.7% for untreated aggregates	n.e.	[78]
Accelerated carbonation (20%) of coarse aggregates	The water absorption of carbonated aggregates was reduced by 19.16%, 21.8%, and 16.3%, for the fractions 5–10 mm, 10–20 mm and 20–25 mm, respectively	n.e.	[79]
Sodium alginate microbial induced calcium carbonate precipitation on RA	32.85% decrease in water absorption after CaCO_3_ precipitation	n.e.	[80]
Accelerated carbonation (20%) of coarse aggregates from different concrete strength classes (C30, C40, and C50)	Up to 20% reduction in water absorption for carbonated RAs from C50-strength concrete (5–10, 10–20, and 20–25 mm) compared to non-treated aggregates	n.e.	[81]
RCAs incorporating air-entraining agents (AEAs) and nano-silica solution (30 wt%)	n.e.	56.4 MPa, 42.7 MPa and 32.9 MPa for 0, 50 and 100% coarse aggregates replacement with 0.05% of AEA (w:c = 0.4)	[82]
RA first immersed in a slurry with a water-to-binder ratio of 0.8, in which 20% of the OPC (ordinary Portland cement) was replaced by FA (fly ash) and SF (silica fume). Next, the coated RAs were immersed in sodium silicate (waterglass, WG) and silicon-based additive solutions	Compared with untreated aggregates, the total pore volume of C30 concrete with RAs with WG and SA decreased by 30.3% and 32.9%, while the average pore diameter decreased by 12.5% and 24.7%, respectively	When the replacement rate of RAs is 50% and 100%, the compressive strengths of recycled concrete with the strength grade of C30 are 36.0 and 29.6 MPa, 15.1% and 30.2% lower than that of the ordinary concrete, respectively.	[83]
Pre-soaked or pre-sprayed RAs in a graphene oxide (GO) solution (0, 0.01, 0.03, and 0.05 wt% of GO with respect to cement)	Compared with untreated aggregates, the total porosity of RAs pre-soaked in a solution with 0.05 wt% of GO decreased by 27.8%. The detrimental pores with an equivalent diameter larger than 50 nm was reduced by 35.5%.	Compressive strength increased by 8.3%, 18.1%, and 30.8% at 7 days, and 9.5%, 14.4% and 23.5% at 90 days, for RAs pre-soaked in a solution with 0.01, 0.03, and 0.05 wt% of GO, respectively, compared to untreated aggregates. With 5 wt% addition of GO, the compressive strength was very close to that of concrete made with NAs (w–binder = 0.55, 100% coarse aggregates replacement).	[84]
Bio-deposition of biogenic silica by means of diatoms	Hydrophobicity close to that observed in nano-silica (50 nm) coatings (50 wt%)	Increase in the compressive strength up to 8% in function of the diatoms’ growth conditions (indoor or outdoor) compared to non-treated aggregates (w:c = 0.59, 50% coarse aggregates replacement)	[85]
Immersion in 2, 5, 10 and 30 wt% NS solutions for 24 h of RA (5–10 and 10–20 mm)	Compared to untreated RA, the water absorption of RAs treated with 2%, 5%, 10%, and 30% NS solution decreased by 18.2%, 21.1%, 22.6%, and 27.6%, respectively; 2% concentration of NS solution is sufficient to consume portlandite of RA	The compressive strengths of concrete made with RAs treated with NS solutions at different concentrations (2%, 5%, 10%, and 30%) were, respectively, 14.5%, 17.2%, 25.9%, and 28.8% higher compared to the concrete manufactured with untreated RA. Further improvement of 5.4%, 6.4%, 5.1%, and 6.9%, respectively, if excess NS not removed from the surface	[86]
Soaking in 3% bacterial solution and 0.1 mol/L calcium acetate solution followed by carbonation with 20% CO_2_ (101 kPa)	4.72% water absorption for the 3% concentrated bacterial solution, compared to 9% for untreated aggregates	n.e.	[87]
MICP based on hydrolyzing urea (best results when the ratio of bacterial solution concentration to urea concentration = 5)	10% decrease in water absorption for treated aggregates with respect to untreated ones	48 MPa compressive strength for the concrete made with treated aggregates (+19.5% with respect to concrete made with untreated aggregates) (w:c = 0.49, 100% coarse aggregates replacement)	[88]
RA soaking in slurries based on OPC and diatomaceous earth (DE) ((OPC + DE):water = 1:2 by weight)	Increase in water absorption with increase in DE in the slurry (up to 20%): from 2.32% for untreated aggregates to 5.15% for 20%DE slurry	20% increase in compressive strength for the concrete with the aggregates soaked in the slurry with 5% DE, with respect to non-treated aggregates (w:c = 0.5, 100% coarse aggregates replacement)	[89]
Carbonation of coarse RA (20% CO_2_) from C40-strength concrete	16.3–21.8% lower water absorption for carbonated aggregates in function of their size (5–10, 10–20, and 20–25 mm). About +160% water absorption with respect to natural aggregates with the same size	The average compressive strength of concrete made with carbonated RAs was 52.47 MPa, compared to 54.37 MPa for the concrete manufactured with natural aggregates and 47.43 MPa for samples with non-treated RAs (w:c = 0.4, 100% coarse aggregates replacement)	[90]
Immersion of coarse RA in an 8% solution of sodium silicate and a 12% solution of silane	8.12% water absorption for RA, compared to 3.83% for treated RA	Compressive strength of concrete made with impregnated RAs increased from 33.75 to 38.09 MPa (+12.86%) with respect to that made of pristine RAs (w:c = 0.5)	[91]
Presoaking for 24 h in a 1.5 wt% NS solution combined with carbonation (99%, 300 kPa) of coarse RA	About 22% reduction in water absorption for treated RA. Limited variations in function of the studied fractions (4.75–7, 7.5–9, and 9.5–12 mm)	Compared to cast samples, the loss ranges in compressive strength of printed specimens were 15.37–19.41% in the X direction, 21.61–25.62% in the Y direction, and 28.65–33.04% in the Z direction, in function of the replacement rates (30, 70, and 100%) (w:c = 0.41–0.61, higher for higher replacement rates)	[92]
Carbonation (20% CO_2_) of RFA in 0.5 M and 1.0 M NaOH solutions	9.1% for fine RAs carbonated at 0.5 M-45 °C, with respect to 10.1% for pristine RA	Mortars prepared with RFAs carbonated at 0.5 M-45 °C for 10 min acquired comparable strength to the reference mortar prepared with RFAs carbonated at 25 °C for 6 h in absence of NaOH. The highest compressive strength was 48.8 MPa, corresponding to an increase of 17.0% with respect to the mortar made with natural sand (binder–water = 0.5, 100% replacement of RFAs)	[93]
Immersion of coarse RA in a commercial crystallizing agent solution for 1 day or 7 days	24.82% water absorption reduction after 1 day of immersion, 58.49% water absorption reduction after 7 days of immersion	46.9 MPa compressive strength for concrete made with 7 days immersed RA, compared to 47.7 MPa for concrete made with NAs (w:c = 0.4, 100% coarse aggregates replacement)	[47]
Immersion of coarse RA (5–10 and 10–25 mm) in a commercial crystalline admixture (CA) solution with waste glass powder (WGP)	Whatever the WGP content, the CA controls the water absorption (≈0.25–0.5% after 11 days)	The compressive strength of the samples decreased with the increasing WGP content, whatever the CA content (1 or 2%) (w–binder = 0.53, 100% coarse aggregates replacement)	[48]
Immersion of RFAs in a commercial crystallizing agent solution	Water penetration under pressure = 6 mm for mortar made with SS, while mortar made with treated RFAs (cured for 15 days) showed a penetration of 9 mm	About 65 MPa compressive strength for 100% replacement of fine aggregates with treated RFAs (CON-Y, cured 15 days), compared to about 55 MPa for mortars made with standard sand	This work

n.e.: not evaluated; X-CT: X-ray computed tomography. All mechanical properties reported in the table were determined after 28 days of curing unless specified.

## Data Availability

The original contributions presented in this study are included in the article. Further inquiries can be directed to the corresponding authors.

## Abbreviations

The following abbreviations are used in this manuscript:

AD	Admixplus
CA	Crystalline admixture
CDW	Construction and demolition waste
DE	Diatomaceous earth
FA	Fly ash
ITZ	Interfacial transition zone
LE	Linear economy
MICP	Microbial induced carbonate precipitation
NA	Natural aggregate
NS	Nano-silica
OPC	Ordinary Portland cement
RA	Recycled aggregate
RCA	Recycled concrete aggregate
RCPI	Rapid chloride permeability index
RFA	Recycled fine aggregate
RS	Recycled sand
SF	Silica fume
SP	Superplasticizer
SS	Standard sand
TBP	Three-point bending
W:c	Water to cement ratio
WG	Waterglass
WGP	Waste glass powder
